# Genetic structure of coexisting wild and managed agave populations: implications for the evolution of plants under domestication

**DOI:** 10.1093/aobpla/plv114

**Published:** 2015-10-03

**Authors:** Carmen Julia Figueredo, Alejandro Casas, Antonio González-Rodríguez, Jafet M. Nassar, Patricia Colunga-GarcíaMarín, Víctor Rocha-Ramírez

**Affiliations:** 1 Instituto de Investigaciones en Ecosistemas y Sustentabilidad, Universidad Nacional Autónoma de México, Campus Morelia, Apartado Postal 27-3 (Santa María de Guido), Morelia, Michoacán 58190, Mexico; 2 Centro de Ecología, Instituto Venezolano de Investigaciones Científicas, Carretera Panamericana km 11, Apdo. 20632, Altos de Pipe, Miranda 1020-A, Venezuela; 3 Centro de Investigación Científica de Yucatán, A.C. Calle 43 No. 130, Colonia Chuburná de Hidalgo, CP 97200 Mérida, Yucatán, México

**Keywords:** *Agave inaequidens*, Agaves, conservation genetics, domestication, genetic structure, genetic variation, microsatellites

## Abstract

Domestication is a continuous evolutionary process guided by humans. This process leads to divergence in characteristics such as behaviour, morphology or genetics, between wild and managed populations. Agaves have been important resources for Mesoamerican peoples since prehistory. Some species are domesticated and others vary in degree of domestication. *Agave inaequidens* Koch is used in central Mexico to produce mescal, and a management gradient from gathered wild and silvicultural populations, as well as cultivated plantations, has been documented. Significant morphological differences were reported among wild and managed populations, and a high phenotypic variation in cultivated populations composed of plants from different populations. We evaluated levels of genetic diversity and structure associated with management, hypothesizing that high morphological variation would be accompanied by high genetic diversity in populations with high gene flow and low genetic structure among managed and unmanaged populations. Wild, silvicultural and cultivated populations were studied, collecting tissue of 19–30 plants per population. Through 10 nuclear microsatellite loci, we compared population genetic parameters. We analysed partition of variation associated with management categories to estimate gene flow among populations. *Agave inaequidens* exhibits high levels of genetic diversity (*He* = 0.707) and moderate genetic structure (*F*_ST_ = 0.112). No differences were found in levels of genetic diversity among wild (*He* = 0.704), silviculturally managed (*He* = 0.733) and cultivated (*He* = 0.698) populations. Bayesian analysis indicated that five genetic clusters best fit the data, with genetic groups corresponding to habitats where populations grow rather than to management. Migration rates ranged from zero between two populations to markedly high among others (*M* = 0.73–35.25). Natural mechanisms of gene flow and the dynamic management of agave propagules among populations favour gene flow and the maintenance of high levels of variation within all populations. The slight differentiation associated with management indicates that domestication is in an incipient stage.

## Abstract (precis)

*Agave inaequidens* is used to produce mescal, and a management gradient from gathered wild, silvicultural and cultivated plantations has been documented. We expected different levels of genetic diversity and structure associated with management. Through 10 nuclear microsatellite loci we compared population genetics parameters and found that *A. inaequidens* exhibits high levels of genetic diversity (*He*=0.707) and moderate genetic structure (*F*_*ST*_=0.112) with no differences among wild and managed populations. Bayesian analysis indicated that genetic clusters best fit with the corresponding habitats where populations grow. Natural mechanisms of gene flow and movement of agave propagules among populations by people explain these patterns.

## Introduction

Domestication is a gradual and continuous process through which plants undergo phenotypic and genetic changes, mainly resulting from artificial selection favouring organisms with features desirable, necessary or interesting to humans (Darwin 1859; Casas *et al*. 2007; Pickersgill 2007). Since Darwin's time (Darwin 1859, 1868), domestication has been a useful model for studying a variety of questions in evolutionary biology (Gepts 2004; McKey *et al*. 2012). Understanding the relationship between domesticated and wild individuals provides valuable opportunities for studying evolutionary processes in nature, especially in areas where the ancestor and descending organisms coexist and are available for comparison (Olsen and Wendel 2013). The most common pattern of domestication involves a reduction of genetic variation in domesticated populations compared with wild ones, mainly due to artificial selection (Sauer 1972; Doebley 1989; Olsen and Wendel 2013). However, some studies in plants have documented that such a trend may vary according to the life history of the plant species analysed, as well as the context of human cultures involved in use and selection of those species (Casas *et al*. 2006, 2007; Vargas-Ponce *et al*. 2009; Parra *et al*. 2010; Aguirre-Dugua *et al*. 2013).

Mesoamerica is a main centre of diversity of useful plant species (Caballero *et al*. 1998). It is also the centre of domestication of nearly 200 economically important plant species worldwide, such as maize, beans, chilli peppers, cocoa, cotton, prickly pears (Vavilov 1951; Harlan 1975) and among them several species of agaves (Gentry 1982; Colunga-GarcíaMarín *et al*. 2007). In addition, numerous native plant species (>800) are at various stages of domestication in traditional farming systems, such as home gardens and other agroforestry systems, most of them still unstudied (Colunga-GarcíaMarín and Zizumbo-Villarreal 1993; Casas *et al*. 1997, 2007; Caballero *et al*. 1998; Blancas *et al*. 2010). In these systems, it is possible to find coexisting wild, semi-domesticated or incipiently domesticated, and fully domesticated plants belonging to a single species (Casas *et al*. 2007). In Mesoamerica, people practice *in situ* management of wild plants in forests, forest patches and agroecosystems, which includes several types of interaction: letting stand, encouraging growth and special care and protection of more favourable plants. These interactions in some cases involve artificial selection, resulting in documentable domestication processes (Colunga-GarcíaMarín and Zizumbo-Villarreal 1993; Casas *et al*. 1996, 2007).

Agaves belong to the family Asparagaceae, which comprises 9 genera and ∼300 species (Eguiarte *et al*. 2000; Rocha *et al*. 2006). The genus *Agave* is relatively recent, about 10 million years (my) old, with >160 species, ∼75 % of them occurring in Mexico, where it has shown a high adaptive radiation in diverse ecosystems (Eguiarte *et al*. 2000). Among the most meaningful adaptations of the genus, several authors (Rocha *et al*. 2006; Eguiarte and Souza 2007) have highlighted the morphological and physiological defences against predators, and substantial diversity of reproductive mechanisms and pollinators. Agaves have high chromosome number and large genome size, nearly 64.3 % of species being polyploid (Goldblatt 1980; Palomino *et al*. 2007), showing different ploidy levels. For instance, for *Agave cupreata* Trel. et Berg., Palomino *et al*. (2012) reported diploids, tetraploids, pentaploids and hexaploids. For *A. tequilana* Weber, Bennett *et al*. (2000), Robert *et al*. (2008) and Simpson *et al*. (2011) recorded that genome size varies from 2940 to 77 458 million base pairs.

In a review considering 22 species of *Agave*, Eguiarte *et al*. (2013) estimated that, on average, genetic diversity of this genus is high and genetic differentiation is moderate compared with other outcrossing monocots (Hamrick and Godt 1996).

About 102 taxa of agaves have been reported as being used in Mexico (Colunga-GarcíaMarín *et al*. 2007; Torres *et al*. 2015*a*), most notably as human food. Before maize was adopted as the main Mesoamerican crop, agaves were a principal source of carbohydrates for peoples of western Mexico and an area that is now the southwest USA, who consumed the stems, leaf bases and flower stalk cooked in stone ovens (Callen 1965; Smith 1986; Hodgson 2001; Zizumbo-Villarreal *et al*. 2013).

At present, flower buds, flower stalk and leaf bases are edible, whereas the sap, called *aguamiel*, is collected for drinking fresh or for preparing the fermented drink called *pulque*. In addition, various distilled beverages (generically called mescal, including the appellations of origin Tequila, Bacanora and Raicilla) are prepared with fermented cooked stems. Other important uses are as a source of fibre, and as fodder. The activity that has gained the highest economic importance in recent decades is the production of mescal, spirit drinks produced from the central corm of the plant. For the production of this beverage, the use of 53 taxa has been recorded in Mexico (Colunga-GarcíaMarín *et al*. 2007; Torres *et al*. 2015*a*), most of them extracted from forests. The selection and harvesting of agave corms are carried out just before sexual reproduction takes place, which poses a high risk for numerous populations of several species (Delgado-Lemus *et al*. 2014).

Only four Mexican *Agave* taxa and two of the USA referred to as crops have been studied in terms of the consequences of divergence in genetic diversity caused by domestication and management practices. Among the species studied are *A. fourcroydes* Lem., which was domesticated by the Pre-Columbian Maya of the Yucatán Peninsula for fibre, and their putative ancestor *A. angustifolia* Haw. (Gentry 1982). Using isozyme markers, Colunga-GarcíaMarín *et al*. (1999) found that *A. fourcroydes* exhibits lower levels of genetic diversity than wild *A. angustifolia* populations, probably due to the predominant asexual reproduction of a clone selected for commercial monoculture plantations since the beginning of the 20th century, in addition to the disappearance of the traditional management. In southern areas of the state of Jalisco, the complex of taxa related to *A. angustifolia* has been used to produce mescal and tequila. Currently, it is possible to find wild varieties, traditionally managed varieties (of the species *A. angustifolia* and *A. rhodacantha* Trel.) and one variety that is predominately found in commercial monoculture plantations (*A. tequilana* var. azul). Using inter simple sequence repeat markers, Vargas-Ponce *et al*. (2009) found that traditional landraces of *A. angustifolia* have genetic diversity levels similar to those recorded in wild populations. In contrast, genetic diversity in *A. tequilana* was markedly low. These trends can be explained as a result of the differences between traditional management and commercial monocultures, and the predominant asexual method used to propagate agave crops. In southeast Arizona, *A. parryi* Engelm. and *A. p.* var*. huachucensis* (Baker) Little were cultivated since prehistory, as sources of food and fibre. A number of relict populations from ancient cultivated areas remain in modern landscapes. Parker *et al*. (2010, 2014) evaluated the genetic divergence between wild and anthropogenic populations (relicts of ancient managed populations) through isozymes and microsatellites. In this case, the genetic diversity in the anthropogenic populations was lower than that of wild populations, a trend that can be explained due to dominance of asexual reproduction in anthropogenic populations.

In the cases mentioned, the general trend observed is that cultivated populations exhibit lower levels of diversity than wild populations. The most frequent explanation for this pattern is that genetic diversity in wild populations has been modelled for millions of years, whereas managed populations most commonly involve a fraction of such variation in time periods much shorter. However, in the case of the complex *A. angustifolia* in southern Jalisco, it is clear that traditional management allows the occurrence of high genetic diversity levels in the crop lands, similar or even higher than in the wild, through the constant let standing or encouraging of plants from wild populations already growing in the crop land, or introducing them to these areas. A similar pattern was also reported for several columnar cacti species (Casas *et al*. 2007; Parra *et al*. 2008, 2010).

*Agave inaequidens* Koch is distributed mainly in pine and pine-oak forests of the Trans-Mexican Volcanic Belt. Historically, this species was consumed as food and its sap extracted for drinking fresh or fermented (Gentry 1982). At present, it is used mainly for producing mescal and fibre. In Michoacán, this species is found in a gradient of management intensity with populations occurring in wild habitats as part of natural forests, but also under silvicultural or *in situ* management, through which people leave some individuals standing when the forest is cleared, and deliberately propagate agaves in the cleared areas in order to increase their population density (Torres *et al*. 2015*b*). In addition, some people cultivate this agave away from its natural habitat (*ex situ* cultivation), by transplanting saplings taken from their wild populations and others produced in seedbeds or nurseries, but this practice is relatively new, no more than 30 years old (Figueredo *et al*. 2014; Torres *et al*. 2015*b*).

Based on previous ethnobotanical and morphological studies, we (Figueredo *et al*. 2014) documented the morphological variation and differential use of variants in populations of *A. inaequidens*. We also documented that cultivated populations are composed of individuals from different wild populations, propagated by both seeds and vegetative propagules (Figueredo *et al*. 2014; Torres *et al*. 2015*b*). This background leads us to hypothesize that the high morphological variation observed in cultivated and managed populations would be accompanied by high genetic diversity, similar to that found in wild populations of this species. Furthermore, given the dynamics of formation of crops of this species, and the relatively short time this activity has been carried out, we expected to record high levels of genetic diversity in both managed *in situ* and cultivated populations. We also expected to document high levels of gene flow among managed and unmanaged populations and low genetic structure among populations.

Our study aimed to (i) assess levels of genetic diversity in populations of *A. inaequidens* within a gradient from low to intensive management; (ii) evaluate the levels of genetic structure and divergence between wild, silvicultural managed and cultivated populations of *A. inaequidens* and (iii) estimate gene flow among populations under different management practices, in order to identify possible sources of the gene pools.

## Methods

### Study species

*Agave inaequidens* is recognized with the common name of ‘maguey alto’, which makes reference to its large size, or ‘maguey bruto’, which makes reference to the caustic property of its tissue, because of the presence of saponins and other secondary metabolites that can cause dermatitis (Gentry 1982). This species is endemic to Mexico, naturally growing throughout the Trans-Mexican Volcanic Belt (Gentry 1982). Its distinctive characteristic is teeth dimorphism (successively one shorter and one larger) along the leaves' edges. It is monocarpic, with yellow flowers, protandrous and xenogamous, with pollination conducted by bats and also diurnal animals (León-Jacinto 2013). Fruits are capsules producing shiny black seeds dispersed by the wind (Gentry 1982; León-Jacinto 2013).

This species has >30 different documented uses (Torres *et al*. 2015*b*). In the past, this agave was more important as food, since its inflorescences and stems were cooked in underground ovens and consumed. Its sap was extracted for drinking fresh or fermented (Gentry 1982). Nowadays, those uses are rather rare, and its main use is mescal production and, in some areas, the extraction of its fibres for manufacturing cords (Valenzuela-Zapata *et al*. 2011).

### Study area

**Figure 1.**
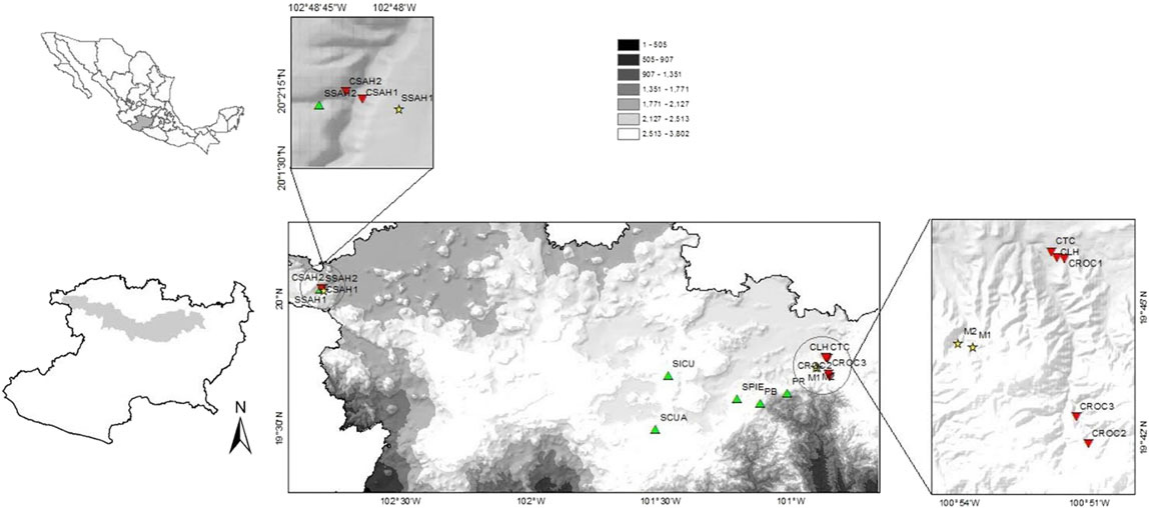
Locations of the 16 populations of *A. inaequidens* studied in Michoacán state. Triangle, wild; square, managed; inverted triangle, cultivated populations.

We sampled a total of 16 populations of *A. inaequidens*: 6 wild, 7 cultivated and the only 3 silvicultural *in situ* managed populations accessible in the state of Michoacán (Fig. 1). Wild populations were growing in pine-oak, oak and subtropical forests. Seven cultivated populations are found growing in orchards together with other species of agaves and fruit trees, where part of the forest was removed for cultivation. Two silvicultural managed populations found in the region grew in secondary forests (natural forest cover has decreased) and one in pastureland (Table 1). We collected tissue of young healthy leaves of 19–30 individuals per population. Tissue samples were kept in silica gel until extraction of total DNA.

**Table 1.** Sampling sites studied for *A. inaequidens* in Michoacán State. The localities were classified according to their category in wild, cultivated and managed. Acronyms of localities and sampling size are shown in parentheses.

Category	Population name and municipality	Acronym (*n*)	Elevation (m)	Vegetation
Wild	Piedra del Indio, Morelia	SPIE (30)	2414	Pine-oak forest dominated by species of *Pinus* and *Quercus*
	Cuanajo, Pátzcuaro	SCUA (30)	2541	Oak forest dominated by *Q. castanea*, *Q. candicans*, *Q. laeta*, *Q. crassipes* and *Q. rugosa*
	Pino Real, Queréndaro	SPR (30)	2323	Pine-oak forest dominated by species of *Pinus* and *Quercus*
	Icuacato, Quiroga	SICU (30)	2475	Oak forest dominated by *Q. castanea*, *Q. candicans*, *Q. laeta*, *Q. crassipes* and *Q. rugosa*
	Pino Bonito, Queréndaro	SPB (30)	2328	Pine-oak forest dominated by species of *Pinus* and *Quercus*
	La Manga Manseña, Sahuayo	SSAH2 (19)	1913	Subtropical scrub dominated by *Euphorbia tanquehuete*, *Bursera fagaroides*, *Eysenhardtia polystachya* and *Ipomea murucoides*. Approximately 100 plants
	Ejido de Parras, Queréndaro	ROC1 (30)	2127	Orchard of 2 ha with fruit trees and other *Agaves* species (*A. angustifolia*, *A. cupreata* and *A. americana*). Approximately 7000 plants about 4 years of being transplanted
Cultivated	Los Alamos, Queréndaro	ROC2 (30)	2691	Orchard 5 ha with fruit trees. Around 5000 plants about 8 years of being transplanted
	El Salto, Queréndaro	ROC3 (30)	2383	Orchard with few fruit trees, species of *Pinus* sp., 4 ha, with pepper crops. Approximately 4000 plants about 11 years after being transplanted
	Traspatio, Queréndaro	CTC (30)	2059	Home gardens, plants about 8 years after being transplanted
	La Huertilla, Queréndaro	CLH (30)	2078	Orchard of ¼ ha growing only *A. inaequidens*, with about 9 years after being transplanted
	Barranca del añil, Sahuayo	CSAH1 (30)	1909	Orchard 5 ha with fruit trees. Approximately 50 plants about 3 years of being transplanted
	Lindero Don Tarsicio, Sahuayo	CSAH2 (20)	1710	Plants arranged as living fences, and mature individuals are used to extract ‘aguamiel’
Managed	Aguacatillos, Indaparapeo	M1 (30)	2508	Secondary vegetation with few oaks. The management in this area is seedlings transplants
				When they are grouped (i.e. aggregate spatial distribution), they are more dispersed
	Salecillo, Indaparapeo	M2 (30)	2486	Same as referred to above
	La Paja, Sahuayo	SSAH1 (30)	1901	Pastureland dominated by *Mimosa* sp. Some individuals aligned as living fence

### DNA extraction, amplification, marker screening and data quality

Total DNA was extracted following the cetyltrimethylammonium bromide protocol for plants (Lefort and Douglas 1999). We used nuclear microsatellites as genetic markers. Eleven different oligonucleotides or primers were assayed, which were originally developed for the studies of *A. parryi*, *A. parryi* var. *huachucensis* and *A. palmeri* (Parker *et al*. 2010, 2014; Lindsay *et al*. 2012).

Polymerase chain reaction (PCR) was conducted using the QIAGEN (www.qiagen.com) Multiplex kit at a final volume of 5 µL, including 2.5 µL of Master Mix (with HotStartaq DNA polymerase, Multiplex PCR buffer, 3 mM MgCl_2_ and deoxynucleotides), 1 mM of each primer, 1.5 µL sterilized water and 0.5–1.0 µL of 50–100 ng µL^−1^ DNA. For all microsatellite loci, amplifications were carried out with a EscoSwiftMaxPro thermal-cycler, using the following protocol: initial heat activation for 15 min at 95 °C, and then 35 cycles of denaturing at 95 °C for 1 min, annealing at 60 °C for 1 min 30 s for all microsatellite loci and extension at 72 °C for 1 min. We included a final step of extension at 72 °C for 7 min. PCR products were mixed with formamide and the size standard Gene Scan LIZ-500 (Applied Biosystems) and denatured for 5 min at 95 °C. PCR products were analysed in a 3130xl Genetic Analyser (Applied Biosystems) sequencer. The resulting electropherograms were analysed using the program Peak Scanner (Applied Biosystems).

Possible genotyping errors due to the presence of null alleles, large alleles or stuttering were identified using the MicroChecker 2.2.3 software (Van Oosterhout *et al*. 2004) with 1000 bootstrap simulations and a confidence interval (CI) of 95 %. Deviations from Hardy–Weinberg Equilibrium (HWE) were examined for all loci in each population using the exact test with Arlequin version 3.11 (Excoffier *et al*. 2005). Deviations from linkage equilibrium (LE) were estimated using Genepop on the Web program with Fisher's method for each pair of loci (Raymond and Rousset 1995).

### Genetic diversity

Number of alleles per locus (*Na*), effective number of alleles (*Ne*) per locus, expected heterozygosity (*He*), unbiased expected heterozygosity (*uHe*) and observed heterozygosity (*Ho*) were estimated using GenAlEx (Peakall and Smouse 2006). Kruskal–Wallis one-way analysis of variance range tests were performed to identify significant differences in levels of diversity according to the management type for each of the estimated parameters.

### Genetic structure

Following Weir (1996), the fixation index (*F*_ST_) was calculated with the program FreeNA using the excluding null alleles method assuming null alleles, with 10 000 bootstrap repetitions (Chapuis and Estoup 2007). The inbreeding coefficient (*F*_IS_) was calculated correcting for null alleles with the INEst program (Chybicki and Burczyk 2009) using the Bayesian model IIM assuming inbreeding. Every run consisted of 10 000 burn-in and 50 000 periods of Markov Chain Monte Carlo simulations (MCMC). The genetic distances (*DC*) of Cavalli-Sforza and Edwards (1967) were estimated for each pair of populations using the including null alleles correction described in Chapuis and Estoup (2007). From a matrix of Nei's genetic distances (*D*), we constructed a dendrogram through the unweighted pair group method with arithmetic mean (UPGMA) method with 1000 bootstrap replicates of the original matrix with TFPGA 1.3 (Miller 1997) and the MEGA program (Tamura *et al*. 2013). Isolation by distance and Mantel tests were examined with isolations by distance webb service (Jensen *et al*. 2005) using Nei's genetic distances.

### Genetic differentiation and genetic flow

The program STRUCTURE version 2.3.4 (Pritchard *et al*. 2000; Falush *et al*. 2003) was used to perform the Bayesian clustering, in which individuals are probabilistically assigned to one of *K* predefined groups to identify the optimal number of genetic groups (Evanno *et al*. 2005). The optimum group number (*K*) was determined varying *K* from 1 to 17, with 10 runs for each *K* value, in order to determine the maximum value of the posterior probability [ln *P*(*K*)]. Every run consisted of 5.0 × 10^4^ burn-in and 10^6^ periods of MCMC repetitions after the burn-in. We used the admixture model with correlated allelic frequencies without prior information. The number of subpopulations was additionally estimated based on the approach of Evanno *et al*. (*2*005) using the software STRUCTURE Harvester (Earl 2011). In order to align the cluster membership coefficients of the 10 structure runs and to graphically display the results, we used the programs CLUMPP version 1.1.2 (Jakobsson and Rosenberg 2007) and Distruct version 1.1 (Rosenberg 2004). Analyses of molecular variance (AMOVAs) were used to test for genetic differences firstly among the populations under the three types of management and then according to the genetic grouping resulting from the Bayesian clustering. For these tests, we used the stepwise mutation models with the program Arlequin version 3.11 (Excoffier *et al*. 2005).

Migration rate (*M* = *m*/*µ*, where *m* is the migration rate per generation and *µ* is the mutation rate) paired in both directions, and Theta (Θ = 4*N*_e_*µ*, where *N*_e_ is the effective size of population) was estimated through MIGRATE-N (Beerli and Felsenstein 1999, 2001), based on maximum likelihood using the Brownian method and a constant mutation rate (*µ*). From the values of *M* and Θ, we estimated the gene flow or number of migrants per population (*Nm*). The effective population size (*Ne*) per population was estimated through an average mutation rate for microsatellites (5 × 10^−4^, Schlötterer 2000; Selkoe and Toonen 2006).

## Results

### Genetic diversity

In total, we successfully amplified 10 microsatellite loci. No evidence for large-allele dropout was detected in any locus using the software Micro-Checker. Null alleles seemed to be present in 14 populations for loci 6 and 7 (APARLC20, APARLC21, Lindsay *et al*. 2012), whereas populations CSAH1 and M1 had no evidence of null alleles. Significant deviations from HWE were recorded associated with heterozygote excess in the wild populations SPIE, SCUA, SICU and SPB; the cultivated populations CROC1, CROC2, SAHC1 and LH and in the silvicultural managed populations M1, M2 and SSAH1. Heterozygote deficiency was recorded in two wild populations (SPR and SSAH2) and three cultivated populations (CSAH2, CROC3 and TC). A global test of LE indicated that genotypes at one pair of loci (APAR2-12 and APARLC28, Lindsay *et al*. 2012) are not independent (*P* ≤ 0.05), but identical results were found in the rest of the analysis when either of these loci was removed.

The *Na* ranged from 4.0 to 8.6, with the highest value found in the wild population SPIE and the lowest value recorded in the wild population SSAH2 and the cultivated population CSAH2 (Table 2). The *Ne* ranged from 2.37 to 5.04, with the highest value found in the wild population SPB. The values of *Ho* ranged from 0.2 to 0.87, with the highest value recorded for the cultivated population CSAH1. *He* ranged from 0.449 to 0.754, with the highest value observed in the cultivated population CLH. For *uHe*, estimates ranged from 0.461 to 0.774; in this case, the managed population M1 had the highest values. An interesting point to emphasize is that the cultivated population CSAH2 exhibited the lowest values in all the diversity parameters estimated (Table 2). Differences in mean values of *Na*, *Ne*, *Ho*, *He* and *uHe* among wild, cultivated and silvicultural managed populations were not statistically significant (*P* > 0.05).

**Table 2.** Summary of genetic diversity estimates (means ± SE) at the population and management levels for *A. inaequidens* based on 10 microsatellite loci. *Na*, mean number of alleles per locus; *Ne*, mean effective number of alleles per locus; *Ho*, mean observed heterozygosity; *He*, mean expected heterozygosity; *uHe*, mean unbiased expected heterozygosity.

Population	*Na*	*Ne*	*Ho*	*He*	*uHe*
SPIE	8.600 ± 1.118	4.750 ± 0.771	0.778 ± 0.082	0.734 ± 0.042	0.747 ± 0.043
SCUA	8.300 ± 1.309	4.594 ± 0.729	0.764 ± 0.066	0.724 ± 0.044	0.738 ± 0.045
SPR	8.000 ± 1.211	4.601 ± 0.727	0.678 ± 0.096	0.731 ± 0.041	0.745 ± 0.042
SICU	8.300 ± 1.055	4.454 ± 0.537	0.742 ± 0.103	0.734 ± 0.039	0.747 ± 0.040
SPB	7.700 ± 1.230	5.041 ± 1.042	0.756 ± 0.067	0.742 ± 0.037	0.756 ± 0.037
SSAH2	4.700 ± 0.731	2.937 ± 0.458	0.311 ± 0.095	0.556 ± 0.077	0.572 ± 0.079
Mean wild population	7.600 ± 0.593	4.396 ± 0.303	0.672 ± 0.074	0.704 ± 0.029	0.717 ± 0.029
CROC1	7.800 ± 0.786	4.540 ± 0.541	0.783 ± 0.062	0.753 ± 0.025	0.767 ± 0.026
CROC3	7.500 ± 0.898	4.393 ± 0.492	0.692 ± 0.091	0.741 ± 0.032	0.754 ± 0.033
CROC2	7.400 ± 1.087	4.474 ± 0.631	0.755 ± 0.071	0.725 ± 0.044	0.739 ± 0.045
CTC	7.400 ± 0.968	4.176 ± 0.455	0.730 ± 0.082	0.733 ± 0.030	0.754 ± 0.029
CSAH1	7.300 ± 0.955	4.184 ± 0.539	0.872 ± 0.044	0.730 ± 0.029	0.743 ± 0.029
CSAH2	4.000 ± 0.683	2.372 ± 0.442	0.200 ± 0.064	0.449 ± 0.086	0.461 ± 0.088
CLH	8.500 ± 0.654	4.434 ± 0.476	0.817 ± 0.069	0.754 ± 0.023	0.769 ± 0.023
Mean cultivated population	7.129 ± 0.544	4.082 ± 0.290	0.693 ± 0.085	0.698 ± 0.041	0.712 ± 0.042
M2	8.100 ± 0.924	4.444 ± 0.469	0.802 ± 0.052	0.750 ± 0.029	0.767 ± 0.029
M1	8.000 ± 0.856	4.408 ± 0.405	0.784 ± 0.057	0.752 ± 0.026	0.774 ± 0.027
SSAH1	7.200 ± 0.646	3.682 ± 0.438	0.828 ± 0.060	0.697 ± 0.031	0.709 ± 0.031
Mean *in situ* managed population	7.767 ± 0.285	4.178 ± 0.248	0.805 ± 0.012	0.733 ± 0.017	0.750 ± 0.020

### Genetic structure and gene flow

The global *F*_ST_ was 0.112 (CI: 0.0783–0.151), indicating moderate genetic structure among populations. The mean *F*_IS_ across loci was significantly different from zero (*F*_IS_ = 0.054; 95 % CI: 0.041–0.069), indicating inbreeding.

**Figure 2.**
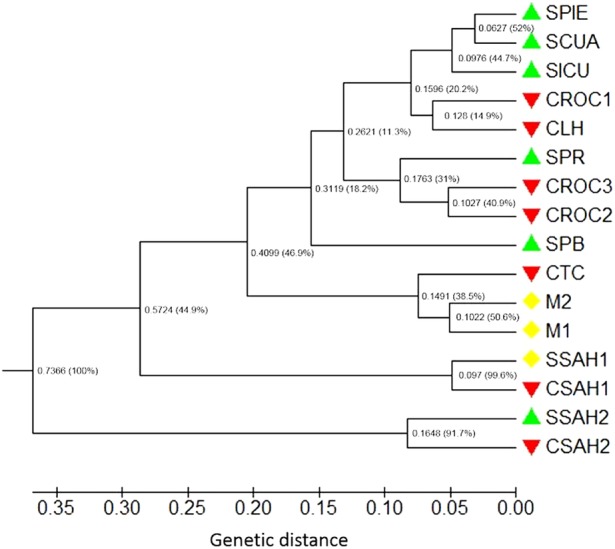
UPGMA cluster based on genetic distances (*D*) estimated among 16 populations of *A. inaequidens*. Numbers are Nei's genetic distances, and numbers in parentheses are the results of one thousand bootstrapping random replicates.

**Figure 3.**
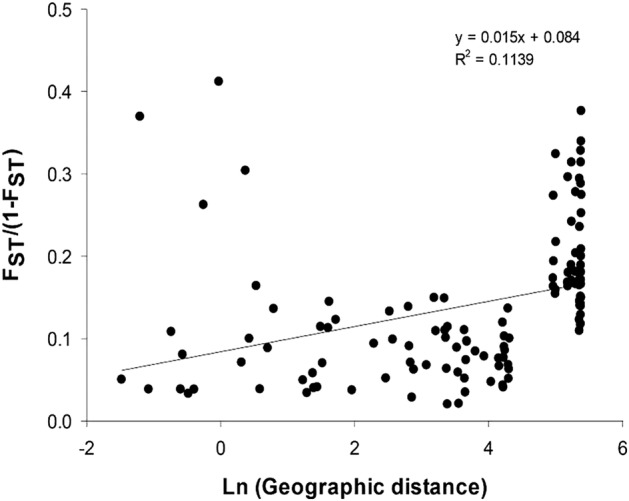
Differentiation among *A. inaequidens* populations. Multilocus estimates of pairwise differentiation (*F*_ST_/[1 − *F*_ST_]) are plotted against the natural logarithm distances (in kilometres).

The UPGMA dendrogram based on genetic distances among populations (Fig. 2) did not cluster populations according to their management type, but several clusters were partially concordant with the geographic location of several populations. Populations SSAH2 and CSAH2, located in the western part of the sampling area, were separated in a group, different from the remaining populations. These populations were also separated in the dendrogram from the SSAH1 and CSAH1. The next group included the four populations of region of Sahuayo in the western part of the study area, separated from the remaining populations. Another group included population CTC and the two silvicultural managed populations M1 and M2. The remaining populations were grouped neither according to their geographic distances nor according to their management category. The regression coefficient of the linear regression analysis of *F*_ST_/(1 − *F*_ST_) on ln of geographic distance (Fig. 3) was positive (*β* = 0.015) and explained 11 % of the variation in *F*_ST_/(1 − *F*_ST_); the association between pairwise in geographic distances and pairwise *F*_ST_/(1 − *F*_ST_) values was significant (Mantel test, *Z* = 6065,23, *r* = 0.62, *P* ≤ 0.0010).

**Figure 4.**
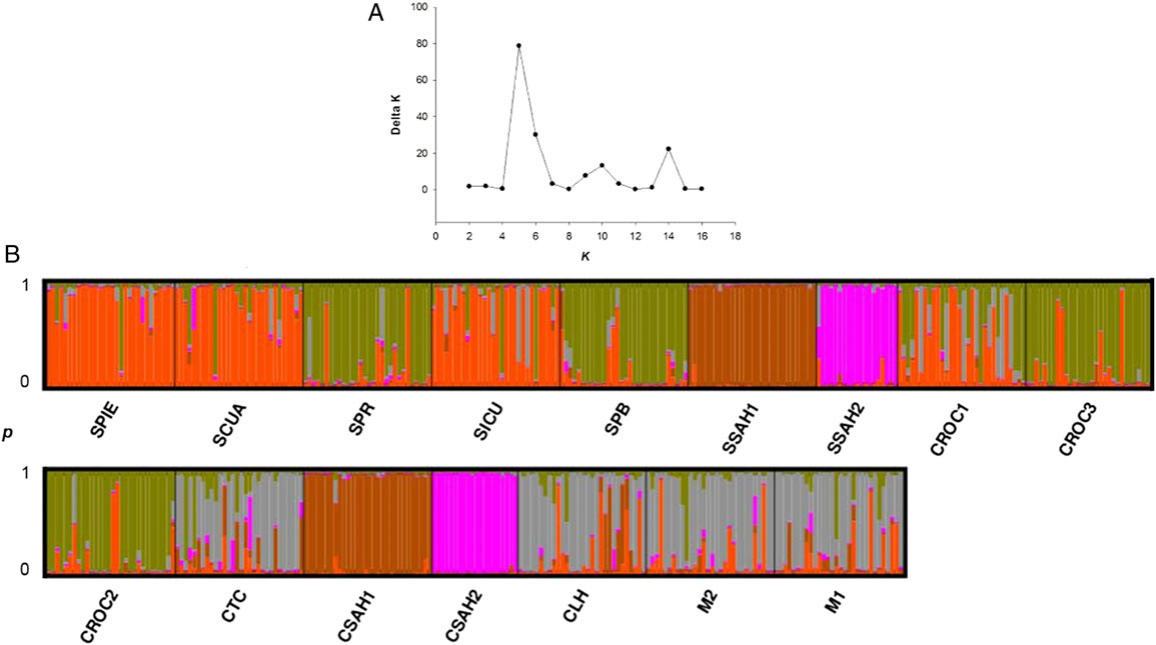
(A) Estimated number of genetic groups (*K*) derived from clustering analysis using STRUCTURE. Delta *K* was calculated using the method described by Evanno *et al*. (2005). (B) Genetic 741 clusters obtained with five groups (*K* = 5). Each individual plant is represented by one vertical line with *K* segments coloured proportionally to their belonging to a genetic cluster.

**Figure 5.**
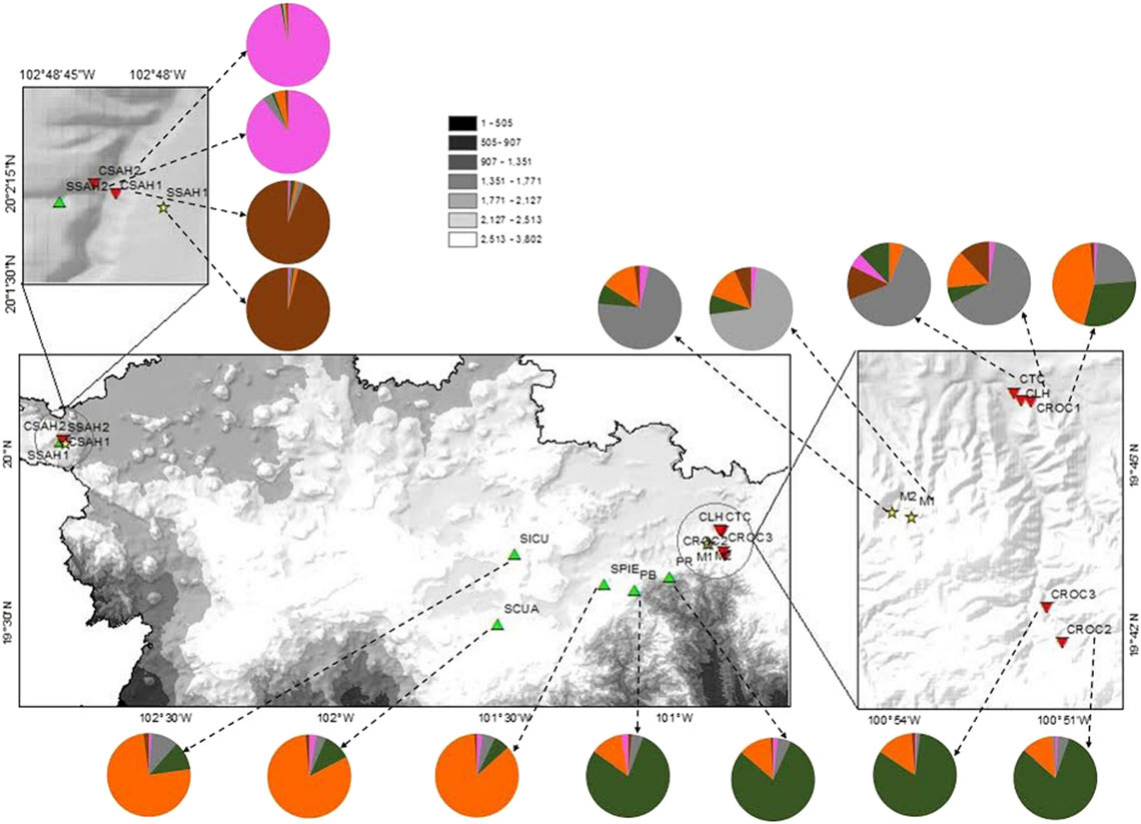
Pie charts showing proportion of ancestry assigned to individuals of each population by Bayesian clustering analysis using STRUCTURE 2.3.4.

The highest value of Δ*K* revealed that the most probable number of genetic groups was *K* = 5 (Fig. 4A). Figure 4B shows the proportion of ancestry of each population and individual plants based on these groups. Three wild populations and one cultivated population (SPIE, SCUA, SICU and CROC1, respectively) had a higher proportion of the orange genetic group. The green genetic group comprised two wild and two cultivated populations (SPR, SPB, and CROC3, CROC2, respectively). The brown genetic group (SSAH1 and CSAH1) and the pink genetic group (SSAH2 and CSAH2) are markedly different, because their members had a very low proportion of ancestry from other genetic groups. The remaining populations were included in the grey genetic group (CLH, CTC, M2 and M1) (Fig. 5). Overall, the grouping pattern under this analysis was very similar to that found through the UPGMA, in which the groups did not correspond to the categories of management but rather are associated with geographic location and other ecological aspects like life-history characteristics.

The AMOVA carried out to test whether populations had hierarchical genetic structure according to the management type categories was not significant (*R*_ST_ = 0; *P* ≥ 0.05). When AMOVA was conducted using the STRUCTURE-defined populations, results indicated that most of the variation occurs among groups (59.32 %; *P* ≤ 0.001, Table 3), followed by the variation within populations (36 %, Table 3). Total differentiation was high and significant among groups (Φ_CT_ = 0.5926; *P* ≤ 0.001) and within populations (Φ_ST_ = 0.6322; *P* ≤ 0.001), whereas differentiation among populations (Φ_SC_ = 0.0972; *P* ≤ 0.001) was moderate but significant.

**Table 3.** Analysis of molecular variance using *R*_ST_ estimates for five genetic groups obtained with STRUCTURE for populations of *A. inaequidens* in Michoacán State. *P ≤ 0.001.

Source of variation	Sum of squares	Components of variance	Percentage of variation	Φ_statistics_
Among genetic groups	1 393 355.745	2140.946	59.259	Φ_CT_ = 0.592*
Among populations within groups	94 475.027	143.053	3.959	Φ_SC_ = 0.097*
Within populations	1 069 513.519	1328.846	36.781	Φ_ST_ = 0.632*
Total	2 557 344.291	3612.845		

The migration rate (*M*) among pairs of populations ranged from 0.703 to 35.25 migrants per generation. The effective population size ranged from 36 in CSAH2 to 709 individuals in the managed population M1 **[see** **Supporting Information—Appendix S1****]**. The gene flow or number of migrants (*Nm*) was below one per generation in all cases considering CSAH2 as the receiver population, and the highest value was 29 migrants from managed populations M2 to M1. In general, the wild populations SICU and SPIE and the cultivated population CROC1 had the highest number of migrants to other populations **[see** **Supporting Information—Appendix S2****]**.

## Discussion

Among all *Agave* species so far examined for genetic diversity, *A. inaequidens* has the highest genetic variation (wild populations averaged *He* = 0.703), showing estimates comparable with those recorded for the wild populations of *A. potatorum* (*He* = 0.549, Félix-Valdez *et al.*, 2015), wild populations of *A. parryi* (*He*= 0.621, Parker *et al*. 2014; *He* = 0.659, Lindsay *et al*. 2012) and wild populations of *A. palmeri* (*He* = 0.550, Lindsay *et al*. 2012), *A. utahensis* (*He* = 0.490; Byers *et al*. 2014) and *A. utahensis* subsp. *kaibabensis* (*He* = 0.408; Byers *et al*. 2014), which have also been studied by using microsatellite loci.

The dynamic movement of propagules among populations of *A. inaequidens* involved in silvicultural management and cultivation could be one of the causes determining high levels of genetic diversity in these populations. Overall high levels of genetic diversity and the moderate levels of genetic structure among populations of *A. inaequidens* are consistent with several life-history traits of this species: predominant sexual reproduction, the outcrossing breeding system (León-Jacinto 2013) and involvement of long-distance pollinators (bats and birds) (León-Jacinto 2013).

Although morphological studies have reported significant phenotypic differences between wild and cultivated populations of *A. inaequidens* (Figueredo *et al*. 2014), this pattern was not consistent with the results of our genetic structure analysis using microsatellite loci. No genetic divergence was detected among populations according to management practice, which indicates that management and artificial selection documented to occur on *A. inaequidens* by other studies (Figueredo *et al*. 2014; Torres *et al*. 2015*b*) have not affected genetic diversity within the species. Artificial selection could be a recent process in this species or not strong enough to have caused genetic differentiation between wild populations and populations exposed to management practices, because human-induced and naturally occurring gene flow may rapidly counteract the effects of artificial selection. Also, it could be that there may be phenotypic plasticity in the genus since phenotypic differences documented previously could be largely influenced by local environments, and therefore, artificial selection would be acting on features that have a low heritability. Or, finally, the neutral markers used are not efficient to identify artificial selection on the phenotypic features evaluated.

### Genetic diversity in *A. inaequidens*

Despite its relatively recent origin (approximately 10 my old), the genus *Agave* has a high adaptive radiation and an active process of speciation, with a diversification rate (0.32 species per my), only comparable with the rosetophyllous *silversword* (0.56 species per my) (Baldwin and Sanderson 1998; Eguiarte and Souza 2007). Often, the highest levels of genetic diversity have been found in the centre of origin, and levels decrease as long as the distance increases until the extremes of the area of distribution (Eckert *et al*. 2008; Figueredo and Nassar 2011; Parra *et al*. 2015). The main area of diversification of the genus *Agave* is central Mexico, where *A. inaequidens* occurs. This fact could help to explain the high levels of genetic diversity of this species.

Polyploidy is a source of genetic variation in plants. Nearly 70 % of *Agave* species are polyploid. We do not discard the possibility that *A. inaequidens* populations have different ploidy levels, similar to what has been recorded in *A. cupreata* (Palomino *et al*. 2012) and *A. parryi* (Parker *et al*. 2014). However, this supposition is yet to be confirmed through cytogenetic and flow cytometric studies. Generally, polyploids show the giant phenotype, which includes large size plants. It is possible that in *A.inaequidens*, polyploidy has an influence on one of the target features of artificial selection in favour of more productive agaves, originally for more edible matter, more *aguamiel* and *pulque* production, and more recently for more efficient mescal production. However, the microsatellites tested showed patterns typical of diploid plants.

Other aspects that can contribute to the high levels of genetic diversity and substantial gene flow are life-history traits (Hamrick *et al*. 1992; Hamrick and Godt 1996). *Agave inaequidens* is self-incompatible, with a xenogamous pollination system, and bat pollinated. All these traits can promote gene flow and high levels of genetic variability within populations (Horner *et al*. 1998; Rocha *et al*. 2006; Eguiarte and Souza 2007; León-Jacinto 2013; Parra *et al*. 2015).

Although, in general, we found high levels of genetic diversity in all studied populations, we identified two populations with comparably lower levels of genetic variation: SSAH2 and CSAH2. Possibly, the differences are associated with anthropogenic effects. The wild population SSAH2 has been under an intensive extraction regime for mescal production for >40 years, suffering significant population decline. In addition to agave extraction, the population has also been affected by livestock raising, because animals may consume saplings and trample seedlings, thus affecting recruitment of new plants (Torres *et al*. 2015*a*), as also documented for *A. potatorum* (Delgado-Lemus *et al*. 2014; Torres *et al*. 2015*a*).

Population CSAH2 is composed of individual agaves that form a live fence, and plants of the fence are used for extracting sap. The process of sap extraction enhances asexual reproduction, forming axillary suckers, and apparently, the new plants forming the fence are clones maintained for several generations (Torres *et al*. 2015*b*). In other species of agave (*A. tequilana*, *A. fourcroydes* and *A. parryi*), it has been similarly reported that the low levels of genetic diversity are due to asexual propagation (Colunga-GarcíaMarín *et al*. 1999; Vargas-Ponce *et al*. 2009; Parker *et al*. 2010, 2014).

### Structure and gene flow among studied localities

We recorded a significant, although slight, pattern of isolation by distance for the set of localities sampled; however, we noticed that this pattern seems mainly driven by distances from the most distant localities. In addition, Bayesian grouping appears to be explained by geographic distance and the type of ecosystem where agaves grow. These results are supported by the AMOVA, indicating that 60 % of the variation occurs among these five genetic groups. Populations from Sahuayo grow in subtropical scrub forest at the western area of Michoacán. These populations are the most genetically distinctive, and there is substantial gene flow among them. These populations could be adapted to a drier and warmer environment. Morphological differences between these populations and populations occurring in oak and pine-oak forests were reported by Figueredo *et al*. (2014).

High levels of historical gene flow were detected among most populations examined, independently of their management type. As referred to above, bat pollination makes possible a long-distance exchange of genes among populations, since *Leptonycteris yerbabuenae* can potentially fly nearly 100 km in one single night (Horner *et al*. 1998). But it is also relevant to indicate that the agave handlers are moving seeds and vegetative propagules from forest to forest, from forests to plantations and among plantations themselves, thus promoting gene flow among populations. The populations with fewer incoming migrants per generation were located in the Sahuayo region, which can be explained by the relatively long distance from other populations.

### Implications for management and conservation

An important aspect associated with management of agaves is that cultivation is conducted mainly in agroforestry systems where sexual and asexual propagules and even plantlets transplanted from different wild and managed sources are maintained together. This management system determines that these managed populations represent important reservoirs of genetic variation. Some handlers sow seeds and then transplant saplings to the forest (Torres *et al*. 2015*a*), which increases the probability of establishment of new agave plants in forests with this management regime. In addition, cultivation of agave together with other crops and other wild plants allows high plant heterogeneity, which can significantly contribute to control pests, an aspect that has been documented for other *Agave* species (Zizumbo-Villarreal *et al*. 2009) and crops (Root 1973). Populations of the western area that grow in drier ecosystems are more vulnerable, particularly because of low population density, continual plant extraction and absence of plans for population recovering. The forms of management developed in the eastern areas should be considered for adoption in the western areas. Exchange of management experiences is possible and necessary. This study identified some effects of the management regimes carried out in the eastern zone of the study area. It is still necessary to evaluate the population genetic patterns of *A. inaequidens* in other regions of Mexico. Particularly interesting would be those areas where the species is used mainly for the production of fibre, which apparently is an activity that has decimated the agave populations, and where no reforestation or cultivation planning has occurred (Valenzuela-Zapata *et al*. 2011).

## Conclusions

*Agave inaequidens* is a species with high levels of genetic diversity along the entire management gradient. Population genetic structure in relation to management practices was moderate, because management is relatively recent, and artificial selection can be rapidly counteracted by high gene flow.

Although we recorded high levels of genetic diversity in general in this species, some populations appear to be at risk, because of low population sizes and levels of genetic diversity, in addition to the continuous removal of individuals for the production of mescal, without a strategy for reforestation or farming.

## Sources of Funding

This research was funded by Posgrado en Ciencias Biológicas, UNAM and the Consejo Nacional de Ciencia y Tecnología CONACYT, the Programa de Apoyo a Proyectos de Investigación e Innovación Tecnológica (PAPIIT IN205111-3 and IN209214) and CONACYT (CB-2013-01-221800).

## Contributions by the Authors

C.J.F., main author, involved in the study design, conducting of field and laboratory work, literature review and general data collection, systematization and analysis, wrote the first draft and concluded the final version of this paper. A.C., main coordinator-supervisor of the research project, contributed with original data and the designing of all the researches providing the information for the current analysis; participated in systematization and analysis of data and reviewed several drafts of the manuscript and concluded the final version this paper. P.C.-G., J.M.N. and A.G.-R. contributed to designing and following progress of the research and fieldwork and data analyses. All authors contributed to writing, reading and approved the final manuscript.

## Conflict of Interest Statement

None declared.

## Supporting Information

The following additional information is available in the online version of this article –

**Appendix S1.** Migration rates (*M* = *m*/*µ*) paired in both directions and parameter Theta (Θ = 4*N*_e_*µ*) for 16 populations of *A. inaequidens.* + = receiving population. Top row of the table shows donor populations, and first column to the left shows populations that receive migrants.

**Appendix S2.** Effective population size (*Ne*) and the number of migrants (*Nm*) for 16 populations of *Agave inaequidens*. + = receiving population. In the table, the donor population is shown in the top of the table and the population that receives is shown in the left of the table.

Additional Information

## References

[PLV114C1] Aguirre-Dugua X, Casas A, Pérez-Negrón E 2013 Phenotypic differentiation between wild and domesticated varieties of *Crescentia cujete* L. and culturally relevant uses of their fruits as bowls in the Yucatan Peninsula, Mexico. Journal of Ethnobiology and Ethnomedicine 9:76 10.1186/1746-4269-9-76 24229087 PMC3842825

[PLV114C3] Baldwin BG, Sanderson MJ 1998 Age and rate of diversification of the Hawaiian silversword alliance (Compositae). Proceedings of the National Academy of Sciences of the USA 95:9402–9406. 10.1073/pnas.95.16.9402 9689092 PMC21350

[PLV114C4] Beerli P, Felsenstein J 1999 Maximum-likelihood estimation of migration rates and effective population numbers in two populations using a coalescent approach. Genetics 152:763–773. 10353916 10.1093/genetics/152.2.763 PMC1460627

[PLV114C5] Beerli P, Felsenstein J 2001 Maximum likelihood estimation of a migration matrix and effective population sizes in *n* subpopulations by using a coalescent approach. Proceedings of the National Academy of Sciences of the USA 98:4563–4568. 10.1073/pnas.081068098 11287657 PMC31874

[PLV114C6] Bennett MD, Bhandol P, Leitch IJ 2000 Nuclear DNA amounts in angiosperms and their modern uses—807 new estimates. Annals of Botany 86:859–909. 10.1006/anbo.2000.1253

[PLV114C7] Blancas J, Casas A, Rangel-Landa S, Moreno-Calles A, Torres I, Pérez-Negrón E, Solís L, Delgado-Lemus A, Parra F, Arellanes Y, Caballero J, Cortés L, Lira R, Dávila P 2010 Plant management in the Tehuacán–Cuicatlán Valley, Mexico. Economic Botany 64:287–302. 10.1007/s12231-010-9133-0

[PLV114C8] Byers C, Maughan PJ, Clouse J, Stewart JR 2014 Microsatellite primers in *Agave utahensis* (Asparagaceae), a keystone species in the Mojave Desert and Colorado Plateau. Applications in Plant Sciences 2:1400047 10.3732/apps.1400047 PMC4162669 25225631

[PLV114C9] Caballero J, Casas A, Cortés L, Mapes C 1998 Patrones en el conocimiento, uso y manejo de plantas en pueblos indígenas de México. Revista Estudios Atacameños 16:181–196.

[PLV114C10] Callen EO 1965 Food habits of some pre-Columbian Mexican Indians. Economic Botany 19:335–343. 10.1007/BF02904803

[PLV114C11] Casas A, Vázquez MC, Viveros JL, Caballero J 1996 Plant management among the Nahua and the Mixtec in the Balsas River Basin, Mexico: an ethnobotanical approach to the study of plant domestication. Human Ecology 24:455–478. 10.1007/BF02168862

[PLV114C12] Casas A, Caballero J, Mapes C, Zárate S 1997 Manejo de la vegetación, domesticación de plantas y origen de la agricultura en Mesoamérica. Boletín de la Sociedad Botánica de México 61:31–47.

[PLV114C13] Casas A, Cruse-Sanders J, Morales E, Otero-Arnaiz A, Valiente-Banuet A 2006 Maintenance of phenotypic and genotypic diversity in managed populations of *Stenocereus stellatus* (Cactaceae) by indigenous peoples in Central Mexico. Biodiversity and Conservation 15:879–898. 10.1007/s10531-004-2934-7

[PLV114C14] Casas A, Otero-Arnaiz A, Peréz-Negrón E, Valiente-Banuet A 2007 *In situ* management and domestication of plants in Mesoamerica. Annals of Botany 100:1101–1115. 10.1093/aob/mcm126 17652338 PMC2759202

[PLV114C15] Cavalli-Sforza LL, Edwards AW 1967 Phylogenetic analysis. Models and estimation procedures. The American Journal of Human Genetics 19:233–257. 6026583 PMC1706274

[PLV114C16] Chapuis MP, Estoup A 2007 Microsatellite null alleles and estimation of population differentiation. Molecular Biology and Evolution 24:621–631. 10.1093/molbev/msl191 17150975

[PLV114C17] Chybicki IJ, Burczyk J 2009 Simultaneous estimation of null alleles and inbreeding coefficients. Journal of Heredity 100:106–113. 10.1093/jhered/esn088 18936113

[PLV114C18] Colunga-Garcíamarín P, Zizumbo-Villareal D 1993 Evolución bajo agricultura y desarrollo sustentable. In: Leff E, Carabias J, eds. Cultura y manejo sustentable de los Recursos Naturales. México, DF: CIIH-UNAM, Miguel Ángel Porrúa, 123–164.

[PLV114C19] Colunga-Garcíamarín P, Coello-Coello J, Eguiarte LE, Piñero D 1999 Isozymatic variation and phylogenetic relationships between henequen (*Agave fourcroydes*) and its wild ancestor *A. angustifoli*a (Agavaceae). American Journal of Botany 86:115–123. 10.2307/2656960 21680351

[PLV114C20] Colunga-Garcíamarín P, Zizumbo-Villarreal D, Martínez-Torres J 2007 Tradiciones en el aprovechamiento de los agaves mexicanos: Una aportación a su protección legal y conservación biológica y cultural. In: Colunga-Garcíamarín P, Eguiarte L, Larqué A, Zizumbo-Villarreal D, eds. En lo ancestral hay futuro: Del tequila, los mezcales y otros agaves. Mexico: CICY-CONACYT-CONABIO-INE, 229–248.

[PLV114C21] Darwin C 1859 The origins of species by means in natural selection or the preservation of favoured races in the struggle for life. London: Wiley. PMC5184128 30164232

[PLV114C22] Darwin C 1868 The variation of animals and plant under domestication. London: John Murray.

[PLV114C23] Delgado-Lemus A, Casas A, Tellez O 2014 Distribution, abundance and traditional management of *Agave potatorum* in the Tehuacán Valley, Mexico: bases for sustainable use of non-timber forest products. Journal of Ethnobiology and Ethnomedicine 10:63 10.1186/1746-4269-10-63 25185769 PMC4237816

[PLV114C24] Doebley JF 1989 Isozymic evidence and the evolution of crop plants. In: Soltis D, Soltis P, eds. Isozymes in plant biology. Portland, OR: Dioscorides Press, 165–191.

[PLV114C26] Earl DA 2011 Structure harvester v0.6. http://taylor0.biology.ucla.edu/struct_harvest/ (10 February 2015).

[PLV114C27] Eckert CG, Samis KE, Lougheed SC 2008 Genetic variation across species’ geographical ranges: the central–marginal hypothesis and beyond. Molecular Ecology 17:1170–1188. 18302683 10.1111/j.1365-294X.2007.03659.x

[PLV114C28] Eguiarte LE, Souza V 2007 Historia natural del Agave y sus parientes: Evolución y Ecología. In: Colunga Garcíamarín P, Larqueé Saavedra A, Eguiarte LE, Zizumbo-Villareal D, eds. En lo ancestral hay futuro: del tequila, los mezcales y otros agaves. Mexico: CICY-CONACYT-CONABIO-INE, 3–21.

[PLV114C29] Eguiarte LE, Souza V, Silva A 2000 Evolución de la familia Agavaceae, filogenia, biología reproductiva y genética de poblaciones. Boletín de la Sociedad Botánica de México 66:131–150.

[PLV114C30] Eguiarte LE, Aguirre-Planter E, Aguirre X, Colín R, González A, Rocha M, Scheinvar E, Trejo L, Souza V 2013 From isozymes to genomics: population genetics and conservation of *Agave* in México. The Botanical Review 79:483–506. 10.1007/s12229-013-9123-x

[PLV114C31] Evanno G, Regnaut S, Goudet J 2005 Detecting the number of clusters of individuals using the software STRUCTURE: a simulation study. Molecular Ecology 14:2611–2620. 10.1111/j.1365-294X.2005.02553.x 15969739

[PLV114C32] Excoffier L, Laval G, Schneider S 2005 Arlequin (version 3.0): an integrated software package for population genetics data analysis. Evolutionary Bioinformatics Online 1:47–50. PMC2658868 19325852

[PLV114C33] Falush D, Stephens M, Pritchard JK 2003 Inference of population structure using multilocus genotype data: linked loci and correlated allele frequencies. Genetics 164:1567–1587. 12930761 10.1093/genetics/164.4.1567 PMC1462648

[PLV114C1a] Félix-Valdez L, Vargas-Ponce O, Cabrera-Toledo D, Casas A, Cibrián-Jaramillo A, de la Cruz-Larios L 2015 Effects of management for mescal production on the diversity and genetic structure of *Agave potatorum* Zucc., in central México. Genetic Resources and Crop Evolution, 10.1007/s10722-015-0315-6.

[PLV114C34] Figueredo CJ, Nassar JM 2011 Population genetics of *Agave cocui*: evidence for low genetic diversity at the southern geographic limit of genus Agave. Journal of Heredity 102:306–314. 10.1093/jhered/esr018 21467156

[PLV114C35] Figueredo CJ, Casas A, Colunga-Garcíamarín P, Nassar JM, González-Rodríguez A 2014 Morphological variation, management and domestication of ‘maguey alto’ (*Agave inaequidens*) and ‘maguey manso’ (*A. hookeri*) in Michoacán, México. Journal of Ethnobiology and Ethnomedicine 10:66 10.1186/1746-4269-10-66 25227277 PMC4177152

[PLV114C36] Gentry S 1982 Agaves of continental North America. Tucson, AZ, USA: The University of Arizona Press.

[PLV114C37] Gepts P 2004 Crop domestication as a long-term selection experiment. Plant Breeding Reviews 24:1–44.

[PLV114C38] Goldblatt P 1980 Polyploidy in angiosperms: monocotyledons. In: Lewis WH, ed. Polyploidy: biological relevance. New York, NY: Plenum Press, 219–239.

[PLV114C39] Hamrick JL, Godt MJW 1996 Effects of life history traits on genetic diversity in plant species. Philosophical Transactions of the Royal Society B: Biological Sciences 351:1291–1298. 10.1098/rstb.1996.0112

[PLV114C40] Hamrick JL, Godt MJW, Sherman-Broyles SL 1992 Factors influencing levels of genetic diversity in woody plant species. New Forests 6:95–124. 10.1007/BF00120641

[PLV114C41] Harlan J 1975 Crops and man. Foundation for modern crop science series. Madison, WI: American Society of Agronomy.

[PLV114C42] Hodgson WC 2001 Taxonomic novelties in American Agave (Agavaceae). Novon 11:410–416. 10.2307/3393152

[PLV114C43] Horner MA, Fleming TH, Sahey CT 1998 Foraging behaviour and energetics of a nectar-feeding bat, *Leptonycteris curasoae* (Chiroptera: Phyllostomidae). Journal of Zoology 244:575–586. 10.1111/j.1469-7998.1998.tb00062.x

[PLV114C44] Jakobsson M, Rosenberg NA 2007 CLUMPP: a cluster matching and permutation program for dealing with label switching and multimodality in analysis of population structure. Bioinformatics 23:1801–1806. 10.1093/bioinformatics/btm233 17485429

[PLV114C45] Jensen JL, Bohonak AJ, Kelley ST 2005 Isolation by distance, web service. BMC Genetics 6:13. v.3.23. 15760479 10.1186/1471-2156-6-13 PMC1079815

[PLV114C46] Lefort F, Douglas GC 1999 Occurrence and detection of triploids by microsatellite analysis. In: Douglas GC, ed. Strategies for improvement of forest tree species. Proceedings of the Teagasc/TDC Symposium on Forest Genetics. Dublin, Ireland: COFORD, 19–35.

[PLV114C47] León-Jacinto A 2013 Aspectos de la fenología, visitantes florales y polinización de Agave inaequidens Koch ssp. inaequidens (Agavaceae) en el estado de Michoacán. Bachellor Thesis, Morelia, Michoacán, Mexico: Universidad Michoacana de San Nicolás de Hidalgo.

[PLV114C48] Lindsay DL, Edwards CE, Jung MG, Bailey P, Lance RF 2012 Novel microsatellite loci for *Agave parryi* and cross-amplification in *Agave palmeri* (Agavaceae). American Journal of Botany 99:e295–e297. 10.3732/ajb.1200033 22753814

[PLV114C49] Mckey D, Elias M, Pujol B, Duputié A 2012 Domestication, evolution, and sustainability. In: Gepts P, Famula TR, Bettinger RL, Brush SB, Damania AB, Mcguire PE, Qualset CO, eds. Biodiversity in agriculture. Cambridge, UK: Cambridge University Press, 377–406.

[PLV114C50] Miller MP 1997 Tools for population genetics analyses (TFPGA) 1.3. A windows program for the analysis of allozymes and molecular population genetic data. Computer software distributed by author.

[PLV114C51] Olsen KM, Wendel JF 2013 A bountiful harvest: genomic insights into crop domestication phenotypes. Annual Review of Plant Biology 64:47–70. 10.1146/annurev-arplant-050312-120048 23451788

[PLV114C52] Palomino G, Martínez J, Méndez I 2007 Variación inter e intraespecífica en especies de Agave por citometría de flujo y análisis de sus cromosomas. In: Colunga-Garcíamarín P, Larqueé Saavedra A, Eguiarte LE, Zizumbo-Villareal D, eds. En lo ancestral hay futuro: del tequila, los mezcales y otros Agaves. Mexico: CICY-CONACYTCONABIO-INE, 41–65.

[PLV114C53] Palomino G, Martínez J, Cepeda-Cornejo V, Pimienta-Barrios E 2012 Nuclear genome size and cytotype analysis in *Agave cupreata* Trel. & Berger (agavaceae). Caryologia 65:281–294. 10.1080/00087114.2012.752915

[PLV114C54] Parker KC, Trapnell DW, Hamrick JL, Hodgson WC, Parker AJ 2010 Inferring ancient *Agave* cultivation practices from contemporary genetic patterns. Molecular Ecology 19:1622–1637. 10.1111/j.1365-294X.2010.04593.x 20345679

[PLV114C55] Parker KC, Trapnell DW, Hamrick JL, Hodgson WC 2014 Genetic and morphological contrasts between wild and anthropogenic populations of *Agave parryi var. huachucensis* in south-eastern Arizona. Annals of Botany 113:939–952. 10.1093/aob/mcu016 24638822 PMC3997635

[PLV114C56] Parra F, Pérez-Nasser N, Lira R, Pérez-Salicrup D, Casas A 2008 Population genetics and process of domestication of *Stenocereus pruinosus* (Cactaceae) in the Tehuacán Valley, México. Journal of Arid Environments 72:1997–2010. 10.1016/j.jaridenv.2008.06.007

[PLV114C57] Parra F, Casas A, Peñaloza-Ramírez JM, Cortés-Palomec AC, Rocha-Ramírez V, González-Rodríguez A 2010 Evolution under domestication: ongoing artificial selection and divergence of wild and managed *Stenocereus pruinosus* (Cactaceae) populations in the Tehuacán Valley, Mexico. Annals of Botany 106:483–496. 10.1093/aob/mcq143 20729372 PMC2924835

[PLV114C58] Parra F, Casas A, Rocha V, González-Rodríguez A, Arias-Montes S, Rodríguez-Correa H, Tovar J 2015 Spatial distribution of genetic variation of *Stenocereus pruinosus* (Otto) Buxb. in Mexico: analysing evidence on the origins of its domestication. Genetic Resources and Crop Evolution 62:893–912. 10.1007/s10722-014-0199-x

[PLV114C59] Peakall R, Smouse PE 2006 GENALEX 6: genetic analysis in Excel. Population genetic software for teaching and research. Molecular Ecology Notes 6:288–295. 10.1111/j.1471-8286.2005.01155.x PMC3463245 22820204

[PLV114C60] Pickersgill B 2007 Domestication of plants in the Americas: insights from Mendelian and molecular genetics. Annals of Botany 100:925–940. 10.1093/aob/mcm193 17766847 PMC2759216

[PLV114C61] Pritchard JK, Stephens M, Donnelly P 2000 Inference of population structure using multilocus genotype data. Genetics 155:945–959. 10835412 10.1093/genetics/155.2.945 PMC1461096

[PLV114C62] Raymond M, Rousset F 1995 GENEPOP (version 1.2): population genetics software for exact tests and ecumenicism. Journal of Heredity 86:248–249.

[PLV114C63] Robert ML, Yoong-Lim K, Hanson L, Sanchez-Teyer F, Bennett MD, Leitch AR, Leitch IJ 2008 Wild and agronomically important *Agave* species (Asparagaceae) show proportional increases in chromosome number, genome size, and genetic markers with increasing ploidy. Botanical Journal of the Linnean Society 158:215–222. 10.1111/j.1095-8339.2008.00831.x

[PLV114C64] Rocha M, Good-Avila SV, Molina-Freaner F, Arita HT, Castillo A, García-Mendoza A, Silva-Montellano A, Gaut BS, Souza V, Eguiarte LE 2006 Pollination biology and adaptive radiation of Agavaceae, with special emphasis on the genus *Agave*. Aliso 22:329–344.

[PLV114C65] Root RB 1973 Organization of a plant-arthropod association in simple and diverse habitats: the fauna of collards (*Brassica oleracea*). Ecological Monographs 43:95–124. 10.2307/1942161

[PLV114C66] Rosenberg NA 2004 DISTRUCT: a program for the graphical display of population structure. Molecular Ecology Notes 4:137–138. 10.1046/j.1471-8286.2003.00566.x

[PLV114C67] Sauer CO 1972 Seeds, spades, hearths, and herds: the domestication of animal and foodstuff. Cambridge: MIT Press.

[PLV114C68] Schlötterer C 2000 Evolutionary dynamics of microsatellite DNA. Chromosoma 109:365–371. 10.1007/s004120000089 11072791

[PLV114C69] Selkoe KA, Toonen RJ 2006 Microsatellites for ecologists: a practical guide to using and evaluating microsatellite markers. Ecology Letters 9:615–629. 10.1111/j.1461-0248.2006.00889.x 16643306

[PLV114C70] Simpson J, Martínez Hernández A, Abraham Juárez MJ, Sandoval SD, Villarreal AS, Romero CC 2011 Genomic resources and transcriptome mining in *Agave tequilana*. Global Change Biology Bioenergy 3:25–36. 10.1111/j.1757-1707.2010.01079.x

[PLV114C72] Smith CE Jr 1986 Preceramic plant remains from GuiláNaquitz. In: Flannery KV, eds. GuiláNaquitz. Archaic foraging and early agriculture in Oaxaca, México. New York: Academic Press, 265–201.

[PLV114C74] Tamura K, Stecher G, Peterson D, Filipski A, Kumar S 2013 MEGA6: Molecular Evolutionary Genetics Analysis version 6.0. Molecular Biology and Evolution 30:2725–2729. 10.1093/molbev/mst197 24132122 PMC3840312

[PLV114C75] Torres I, Casas A, Vega E, Martínez-Ramos M, Delgado-Lemus A 2015a Population dynamics and sustainable management of mescal Agaves in Central Mexico: *Agave potatorum* in the Tehuacán-Cuicatlán Valley. Economic Botany 69:26–41. 10.1007/s12231-014-9295-2

[PLV114C76] Torres I, Blancas J, León A, Casas A 2015b TEK, local perceptions of risk, and diversity of management practices of *Agave inaequidens* in Michoacán, Mexico. Journal of Ethnobiology and Ethnomedicine 11:61 10.1186/s13002-015-0043-1 26242969 PMC4526173

[PLV114C77] Valenzuela-Zapata AG, Lopez-Muraira I, Gaytán MS 2011 Traditional knowledge, *Agave inaequidens* (Koch) conservation, and the charro lariat artisans of San Miguel Cuyutlán, Mexico. Ethnobiology Letters 2:72–80. 10.14237/ebl.2.2011.24

[PLV114C78] Van Oosterhout C, Hutchinson WF, Wills DPM, Shipley P 2004 MICRO-CHECKER: software for identifying and correcting genotyping errors in microsatellite data. Molecular Ecology Notes 4:535–538. 10.1111/j.1471-8286.2004.00684.x

[PLV114C79] Vargas-Ponce O, Zizumbo-Villarreal D, Martínez-Castillo J, Coello-Coello J, Colunga-Garcíamarín P 2009 Diversity and structure of landraces of Agave grown for spirits under traditional agriculture: a comparison with wild populations *of A. angustifolia* (Agavaceae) and commercial plantations of *A. tequilana*. American Journal of Botany 96:448–457. 10.3732/ajb.0800176 21628200

[PLV114C80] Vavilov NI 1951 The origin, variation, immunity and breeding of cultivated plants. Chronica Botanica 13:1–366.

[PLV114C81] Weir BS 1996 Genetic data analysis II: methods for discrete population genetic data. Sunderland, MA: SinauerAssoc., Inc.

[PLV114C82] Zizumbo-Villarreal D, Colunga-Garcíamarín P, Vargas-Ponce O, Rosales-Adame JJ, Nieto-Olivares RC 2009 Tecnología agrícola tradicional en la producción de vino mezcal (mezcal y tequila) en el sur de Jalisco, México. Revista de Geografía Agrícola 42:65–82.

[PLV114C83] Zizumbo-Villarreal D, Vargas-Ponce O, Rosales-Adame JJ, Colunga-García-Marín P 2013 Sustainability of the traditional management of Agave genetic resources in the elaboration of mezcal and tequila spirits in western Mexico. Genetic Resources and Crop Evolution 60:33–47. 10.1007/s10722-012-9812-z

