# Supplementary material for: Genetic structure of coexisting wild and managed agave populations: implications for the evolution of plants under domestication
Source: AoB Plants. 2015 Oct 3;7:plv114. doi: 10.1093/aobpla/plv114 (PMC4641209; doi:10.1093/aobpla/plv114)
Supplement: Additional Information [file supp_7_plv114_index.html]

Genetic structure of coexisting wild and managed agave populations: implications for the evolution of plants under domestication — Genetic structure of coexisting wild and managed agave populations: implications for the evolution of plants under domestication — Additional Information 

# Genetic structure of coexisting wild and managed agave populations: implications for the evolution of plants under domestication

## Additional Information

Additional Information

- Additional Information - Docx file
