# Supplementary material for: Genetic structure of coexisting wild and managed agave populations: implications for the evolution of plants under domestication
Source: AoB Plants. 2015 Oct 3;7:plv114. doi: 10.1093/aobpla/plv114 (PMC4641209; doi:10.1093/aobpla/plv114)
Supplement: Additional Information [file supp_plv114_plv114supp.docx]

**Appendix 1.** Migration rates **(***M* = *m*/μ ) paired in both directions and parameter Theta (Θ = 4*N_e_* μ) for 16 population of *Agave inaequidens.* + = receiving population. Top row of the table shows donor populations, first column to the left show populations that receive migrants.

|  | **Theta** | **SPIE, +** | **SCUA, +** | **SPR, +** | **SICU, +** | **SPB, +** | **SSAH1, +** | **SSAH2, +** | **CROC1, +** | **CROC3, +** | **CROC2, +** | **CTC, +** | **CSAH1, +** | **CSAH2, +** | **CLH, +** | **M2,** | **M1, +** |
| --- | --- | --- | --- | --- | --- | --- | --- | --- | --- | --- | --- | --- | --- | --- | --- | --- | --- |
| **SPIE** | 0.873 | - | 26.141 | 7.502 | 17.991 | 17.779 | 3.369 | 6.043 | 16.490 | 9.665 | 5.876 | 6.074 | 2.789 | 3.673 | 5.421 | 3.949 | 11.632 |
| **SCUA** | 0.983 | 24.620 | - | 17.952 | 8.289 | 19.125 | 5.263 | 4.466 | 17.635 | 10.351 | 6.027 | 5.405 | 7.395 | 2.903 | 13.352 | 3.260 | 10.376 |
| **SPR** | 0.985 | 9.507 | 11.835 | - | 12.463 | 12.903 | 5.013 | 5.471 | 7.561 | 14.881 | 14.101 | 1.935 | 10.113 | 2.903 | 5.099 | 4.036 | 10.840 |
| **SICU** | 1.108 | 15.884 | 8.503 | 13.570 | - | 13.736 | 8.990 | 4.121 | 25.191 | 9.167 | 16.036 | 9.489 | 6.536 | 5.243 | 10.073 | 4.960 | 16.540 |
| **SPB** | 1.297 | 19.532 | 15.854 | 15.131 | 12.330 | - | 0.735 | 3.118 | 5.540 | 14.588 | 5.235 | 8.052 | 6.891 | 3.874 | 3.777 | 11.465 | 6.589 |
| **SSAH1** | 0.380 | 3.970 | 2.919 | 2.389 | 9.326 | 0.943 | - | 1.137 | 6.597 | 2.047 | 4.279 | 5.270 | 10.356 | 2.037 | 4.929 | 3.450 | 4.138 |
| **SSAH2** | 0.235 | 6.249 | 5.226 | 5.008 | 6.362 | 4.869 | 3.552 | - | 3.053 | 3.399 | 3.291 | 5.941 | 7.509 | 14.569 | 13.389 | 2.740 | 5.058 |
| **CROC1** | 1.069 | 14.319 | 12.594 | 8.860 | 24.333 | 5.519 | 3.858 | 5.116 | - | 15.827 | 22.685 | 6.318 | 6.246 | 3.114 | 11.694 | 11.942 | 6.275 |
| **CROC3** | 0.479 | 11.475 | 6.682 | 12.372 | 6.991 | 10.920 | 2.890 | 4.592 | 18.652 | - | 7.246 | 3.400 | 7.980 | 3.280 | 4.943 | 3.857 | 6.216 |
| **CROC2** | 0.783 | 5.526 | 3.376 | 14.298 | 13.810 | 5.018 | 2.022 | 2.957 | 23.044 | 6.896 | - | 5.326 | 5.142 | 3.456 | 11.804 | 4.344 | 1.870 |
| **CTC** | 0.291 | 7.233 | 3.898 | 5.575 | 14.357 | 3.507 | 9.480 | 9.209 | 8.938 | 4.501 | 7.716 | - | 7.992 | 8.986 | 31.228 | 13.973 | 25.794 |
| **CSAH1** | 0.302 | 3.636 | 9.105 | 8.227 | 5.228 | 7.968 | 16.519 | 7.564 | 4.962 | 7.733 | 4.403 | 7.558 | - | 4.813 | 4.618 | 6.783 | 5.053 |
| **CSAH2** | 0.073 | 3.422 | 2.901 | 5.431 | 6.993 | 3.869 | 2.604 | 15.698 | 2.232 | 2.221 | 2.870 | 7.811 | 3.571 | - | 8.619 | 4.389 | 4.082 |
| **CLH** | 0.357 | 8.047 | 7.853 | 5.616 | 12.167 | 6.634 | 4.200 | 14.696 | 5.322 | 9.223 | 11.623 | 35.249 | 6.690 | 4.395 | - | 7.814 | 14.062 |
| **M2** | 0.202 | 7.545 | 5.409 | 7.012 | 6.029 | 10.070 | 2.781 | 6.279 | 10.189 | 4.100 | 11.816 | 10.308 | 9.836 | 5.244 | 8.604 | - | 25.243 |
| **M1** | 1.418 | 10.331 | 9.593 | 10.238 | 13.111 | 10.969 | 9.907 | 5.239 | 7.945 | 5.308 | 3.357 | 19.680 | 4.533 | 1.063 | 11.145 | 20.642 | - |

**Appendix 2.** Effective population size (*Ne*) and the Number of migrants *(Nm*) for 16 population of *Agave inaequidens*. + = receiving population. In the table shown in the top of the table the donor population and to the left of the table the population that receives

|  | ***Ne*** | **SPIE, +** | **SCUA, +** | **SPR, +** | **SICU, +** | **SPB, +** | **SSAH1, +** | **SSAH2, +** | **CROC1, +** | **CROC3, +** | **CROC2, +** | **CTC, +** | **CSAH1, +** | **CSAH2, +** | **CLH, +** | **M2,** | **M1, +** |
| --- | --- | --- | --- | --- | --- | --- | --- | --- | --- | --- | --- | --- | --- | --- | --- | --- | --- |
| **SPIE** | 437 | - | 22.826 | 6.551 | 15.709 | 15.525 | 2.942 | 5.277 | 14.399 | 8.439 | 5.131 | 5.304 | 2.435 | 3.207 | 4.734 | 3.448 | 10.157 |
| **SCUA** | 492 | 24.212 | - | 17.655 | 8.152 | 18.808 | 5.175 | 4.392 | 17.343 | 10.179 | 5.927 | 5.315 | 7.272 | 2.855 | 13.130 | 3.206 | 10.204 |
| **SPR** | 493 | 9.367 | 11.660 | - | 12.279 | 12.713 | 4.939 | 5.390 | 7.449 | 14.661 | 13.893 | 1.907 | 9.963 | 2.860 | 5.024 | 3.976 | 10.680 |
| **SICU** | 554 | 17.592 | 9.417 | 15.030 | - | 15.213 | 9.957 | 4.564 | 27.900 | 10.153 | 17.761 | 10.510 | 7.239 | 5.807 | 11.157 | 5.494 | 18.319 |
| **SPB** | 648 | 25.325 | 20.556 | 19.618 | 15.987 | - | 0.953 | 4.043 | 7.183 | 18.915 | 6.787 | 10.441 | 8.935 | 5.023 | 4.897 | 14.865 | 8.544 |
| **SSAH1** | 190 | 1.508 | 1.109 | 0.908 | 3.543 | 0.358 | - | 0.432 | 2.506 | 0.778 | 1.626 | 2.002 | 3.935 | 0.774 | 1.873 | 1.311 | 1.572 |
| **SSAH2** | 118 | 1.472 | 1.231 | 1.179 | 1.498 | 1.147 | 0.836 | - | 0.719 | 0.800 | 0.775 | 1.399 | 1.768 | 3.431 | 3.153 | 0.645 | 1.191 |
| **CROC1** | 535 | 15.309 | 13.466 | 9.473 | 26.017 | 5.901 | 4.125 | 5.470 | - | 16.922 | 24.254 | 6.755 | 6.678 | 3.330 | 12.503 | 12.768 | 6.709 |
| **CROC3** | 240 | 5.498 | 3.202 | 5.928 | 3.350 | 5.232 | 1.385 | 2.200 | 8.937 | - | 3.472 | 1.629 | 3.823 | 1.572 | 2.368 | 1.848 | 2.978 |
| **CROC2** | 391 | 4.324 | 2.642 | 11.189 | 10.807 | 3.927 | 1.583 | 2.314 | 18.033 | 5.397 |  | 4.168 | 4.024 | 2.705 | 9.237 | 3.399 | 1.463 |
| **CTC** | 146 | 2.107 | 1.136 | 1.624 | 4.182 | 1.022 | 2.762 | 2.683 | 2.604 | 1.311 | 2.248 |  | 2.328 | 2.618 | 9.097 | 4.070 | 7.514 |
| **CSAH1** | 151 | 1.098 | 2.749 | 2.484 | 1.579 | 2.406 | 4.988 | 2.284 | 1.498 | 2.335 | 1.329 | 2.282 |  | 1.453 | 1.394 | 2.048 | 1.526 |
| **CSAH2** | 36 | 0.249 | 0.211 | 0.394 | 0.508 | 0.281 | 0.189 | 1.140 | 0.162 | 0.161 | 0.208 | 0.567 | 0.259 |  | 0.626 | 0.319 | 0.297 |
| **CLH** | 178 | 2.869 | 2.800 | 2.003 | 4.338 | 2.365 | 1.498 | 5.240 | 1.898 | 3.289 | 4.145 | 12.569 | 2.386 | 1.567 |  | 2.786 | 5.014 |
| **M2** | 101 | 1.527 | 1.095 | 1.419 | 1.220 | 2.038 | 0.563 | 1.271 | 2.062 | 0.830 | 2.392 | 2.086 | 1.991 | 1.061 | 1.742 |  | 5.110 |
| **M1** | 709 | 14.646 | 13.600 | 14.515 | 18.588 | 15.552 | 14.045 | 7.428 | 11.263 | 7.525 | 4.759 | 27.901 | 6.426 | 1.507 | 15.800 | 29.264 |  |
